# Post thrombolytic resolution of ST elevation in STEMI patients

**DOI:** 10.12669/pjms.321.8974

**Published:** 2016

**Authors:** Sameer Saleem, Adnan Khan, Ihtesham Shafiq

**Affiliations:** 1 Dr. Sameer Saleem, MBBS, Department of Medicine, Khyber Medical College, Peshawar, Pakistan; 2 Dr. Adnan khan, Final Year Students (MBBS), Rehman Medical College, Peshawar – Pakistan; 3 Dr. Ihtesham Shafiq, Final Year Students (MBBS), Rehman Medical College, Peshawar – Pakistan

**Keywords:** ST elevation, Myocardial infarction, Diabetes, Streptokinase

## Abstract

**Objective:**

To study the effect of timing of thrombolytic therapy, cardiac risk factors and site of infarction on S.T. resolution post thrombolysis in STEMI patients

**Methods:**

This was a descriptive hospital based study conducted at the Hayatabad Medical Complex Peshawar. The duration of our study was 5 months from February 2015 to June 2015. Diagnosis of STEMI in symptomatic patients was based on the ECG recognized. Definition of Myocardial Infarction. Time from onset of chest pain to presentation of patients in emergency was noted through history of patients along with time of streptokinase. ECG recordings of patients were taken upon presentation in Emergency. Serial ECG monitoring was done after administration of Streptokinase (SK). ST resolution was observed in the lead with the maximum ST elevation. Data were presented as frequencies and percentages, chi square test was applied.

**Results:**

Among 83 patients with STEMI 50.6% were males and 49.4% were females with the age group range of 30-83 years. Fifty nine patients (71.08%) with STEMI underwent thrombolysis within 12 hours of onset of chest pain while 24 patients (28.92%), underwent thrombolysis after 12 hours of onset of chest pain. Out of the 59 patients who received thrombolytic therapy before 12 hours, 43 (72.88%)completely resolved, while those who received thrombolytic therapy after 12 hours none of them completely resolved as per ECG findings. By applying chi-square test it gives us value of 36.470, and p-value <0.001. In our study 28 patients were diabetic and out of these six (21.43%) completely resolved as per ECG post thrombolysis, 9 (32.14%) partially resolved and 13 (46.43%) failed to resolve. On the other hand, in non-diabetics out of 55, 37 (67.27%) completely resolved, 12 (21.82%) partially resolved and 6 (10.91%) failed to resolve. Among 61 hypertensive, 26 (42.62%) had complete resolution and in 22 who were non-hypertensive, 17 (77.27%)had complete resolution on ECG. Hyperlipidemia and site of infarction didn’t have statistically significant effect on the resolution of ECG post thrombolysis in STEMI patients.

**Conclusion:**

Patients with diabetes, hypertension and those who receive thrombolysis after 12 hours of onset of chest pain respond poorly to thrombolytic therapy as per ECG findings whereas hyperlipidemia and site of infarction don’t affect the response of STEMI patients to thrombolysis.

## INTRODUCTION

When there is occlusion of an epicardial coronary artery, ST elevation is shown on ECG which is electrical manifestation of the pathophysiological changes and is known as ST-Elevation Myocardial Infarction (STEMI).^1^ Acute myocardial infarction can be defined by taking into account clinical, biochemical, electrographic and pathological characteristics.

Diagnostic tool for ST segment elevation Myocardial infarction (STEMI) is Electrocardiogram (ECG) and therefore it should be done immediately on hospital admission.^2^ In Pakistan, cardiac failure is the commonest complication of acute myocardial infarction.^3^

In the United States coronary heart disease is the leading cause of death with myocardial infarction as one of its presentation. In a study done in 2006, ST elevation was found in one quarter to one third of 1.2 million Americans that had myocardial infarction.^4^ In Pakistan one in five middle-aged adults may have underlying coronary artery disease that can lead to myocardial infarction.^5^

Despite better outcomes with early coronary artery reperfusion for the treatment of MI, mortality from acute myocardial infarction still remains significant and the incidence of heart failure is increasing. Unsuccessful thrombolysis leading to adverse events is usually seen in patients who are not treated early.^6^,^7^ Hence in the treatment of acute myocardial infarction, early thrombolysis has become the established fact.^8^

Thrombus causes closure of vessels in acute myocardial infarction and compromises flow in unstable angina. Percutaneous intervention is an option for managing acute coronary syndrome and it is associated with higher patency rates of coronary arteries than medical therapy in patients with ST-elevation myocardial infarction (STEMI).^9^ In case of ST elevation myocardial infarction, post thrombolytic analysis of ST segment resolution on ECG offers cost effective solution to assess coronary reperfusion.^10^

Successful epicardial vessel thrombolysis is necessary for better prognosis, but the outcome more strongly correlates with the micro vascular flow. ST segment on ECG is therefore a better indicator of prognosis, which cannot be assessed on the basis of cardio angiogram alone.^11^,^12^

This is the first study done in Peshawar showing frequency of STEMI patients with resolving of ECG changes after receiving thrombolytic therapy. It also compares the resolution of ECG changes in MI involving the site of myocardium.

## METHODS

This was a descriptive hospital based study conducted at Hayatabad medical complex (HMC), Peshawar. The duration of our study was 5 months from February 2015 to June2015. The study included data of 83 diagnosed cases of ST elevation Myocardial Infarction. Diagnosis of STEMI in symptomatic patients was based on the ECG criteria. The established criteria of Myocardial Infarction which defines STEMI as new ST elevation at the J point in at least 2 contiguous leads of ≥2 mm (0.2 mV) in men or ≥1.5 mm (0.15 mV) in women in leads V2–V3 and/or of ≥1 mm (0.1 mV) in other contiguous chest leads or the limb leads.

### Inclusion criteria

Admission to the coronary care unit with chest pain, patient’s age more or equal to 30 years and ECG showingST elevation myocardial infarction.

### Exclusion criteria

Past Q-wave myocardial infarction, severe cardiac failure, systolic blood pressure <90 mm Hg, acute myocardial infarction within the preceding seven days, moderate to severe valvular heart disease, known allergy to any medication, and contraindications to use of streptokinase administration within the previous 6 months, allergy to the drug, surgery or cerebrovascular accident within previous 6 weeks, warfarin therapy, active peptic ulcer disease, bleeding disorders and uncontrolled hypertension. A detailed history was taken, particularly of age, sex, occupation, address, history of smoking, diabetes mellitus, hypertension, hyperlipidemia and family history of ischemic heart disease.

Time from onset of chest pain to presentation of patient in emergency was noted through history of patient. The door-to-needle time at the hospital is 30 minutes. ECG recordings of patients were taken upon presentation in Emergency. ST elevation was recorded in millimeters from the lead in which maximum elevation was observed. ECG monitoring was done after administration of Streptokinase (SK). ST resolution was observed in the lead with the maximum ST elevation. Complete resolution was defined as a reduction of >70%, partial resolution as a reduction of 30% to 70%, and no resolution as reduction of <30% in ST elevation 180 minutes post thrombolysis.

All data was analyzed by SPSS (statistical package for Social Sciences) version 20.0 for windows. Continuous data were displayed as the mean±standard deviation, while the categorical and nominal data were presented as frequencies and percentages and chi square test was applied.

## RESULTS

Among 83 patients with STEMI 42(50.6%) were male and 41(49.4%) were female with the age group range of 30-83 years means of 52.93±13.30. Baseline characteristics are shown in Table-I.

**Table-I:** Showing base line characteristic of the patients with STEMI (n=83)

Variable	Frequency (n=83)	Percentage (%)
*Gender*		
Male	42	50.6
Female	41	49.4
*Smoking*		
Never used	35	42.2
Current user	30	36.1
Ex-smoker	18	21.7
*Exercise*		
Yes	23	27.7
No	60	72.3
*Diabetic status*		
Yes	28	33.7
No	55	66.3
*Hypertension status*		
Yes	61	73.5
No	22	26.5
*Dyslipidemia*		
Yes	35	42.2
No	48	57.8

Regarding family history out of 83 patients, 65(78.3%) had diabetes and/or hypertension in their family while rest of 18 (21.7%) didn’t have family history of DM and/or HTN. 49(59%) had ischemic heart disease in their family while 34 (41%) didn’t have. Table-II

**Table-II:** Family history of the patients with STEMI.

	Frequency (n=83)	Percentage (%)
*Diabetes and HTN*		
Yes	65	78.3
No	18	21.7
*Ischemic heart disease*		
Yes	49	59.0
No	34	41.0

**Fig.1:**
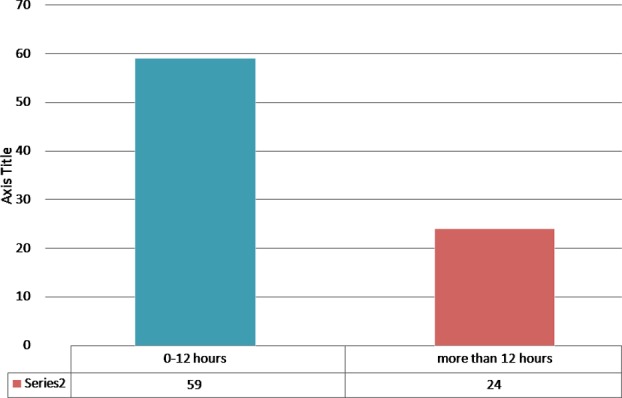
Showing time lapse between chest pain and thrombolytic given.

Fifty nine (71.08%) patients with STEMI were given thrombolytic therapy within 12 hours of onset of chest pain while 24(28.91%) were given thrombolytic therapy after 12 hours of onset of chest pain. Fig.1.

**Fig.2:**
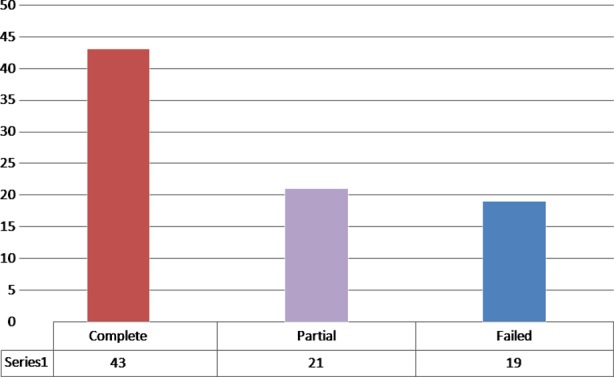
Showing resolution of ECG After thrombolytic.

On ECG 33(39.8%) had anterior MI, 28(33.7%) had inferior MI, 12(14.5%) had lateral MI, 10(12%) had inferior and right ventricular MI. Table-III. After thrombolytic was given, 43 (51.81%) had complete resolution, 21 (25.30%) had partial resolution and 19 (22.89%) failed to resolve as per ECG. Fig.2

**Table-III:** Site of infarction on ECG.

Variable	Frequency (n=83)	Percentage (%)
Anterior MI (V1-V6)	33	39.8
Inferior MI (11,111,aVF)	28	33.7
Lateral MI (1, aVL,V5,V6)	12	14.5
Inferior + Rt ventricular MI (II, III, aVF+V4R)	10	12.0

Total	83	100

## DISCUSSION

Enormous progress has been made in the previous decade for managing acute myocardial infarction. The treatment of myocardial infarction is now based on scientific principles, validated by large well, controlled randomized clinical trials.

One of the well recognized and effective treatments apart from percutaneous intervention for STEMI is Thrombolysis. The aim of thrombolysis in STEMI is early and complete reperfusion. In our study, 78.3% patients with STEMI had family history of hypertension and/or diabetes and 59% patients had family history of ischemic heart disease.

**Table-IV:** Comparison of ECG resolution with other variables.

	Complete (n=43)	Partial (n=21)	Failed (n=19)	Total (n=83)
***Time lapse b/w chest pain & admission***				
Within 12 hours	43	9	7	59
After 12 hours	0	12	12	24
* Chi square = 36.470, degree of freedom = 2, p-value = <0.001*				
***Diabetic status***				
Diabetic	6	9	13	28
Non diabetic	37	12	6	55
*Chi square = 18.535, degree of freedom = 2, p-value = <0.001*				
***Hypertension status***				
Hypertensive	26	19	16	61
Non-hypertensive	17	2	3	22
*Chi square = 7.976, degree of freedom = 2, p-value = 0.019*				
***Dyslipidemia***				
Dislipedimaic	18	12	5	35
Non dyslipidemia	25	9	14	48
*Chi square = 3.891, degree of freedom = 2, p-value = 1.43*				
***Site of Infarction***				
Anterior MI (V1-V6)	16	10	7	33
Inferior MI(11,111,aVF)	14	7	7	28
Lateral MI(1,aVL,V5,V6)	6	2	4	12
Inferior +Rt ventricular MI (II, III, aVF+V4R)	7	2	1	10
*Chi square = 2.919, degree of freedom = 2, p-value = 8.19*				

Certain risk factors predispose to acute myocardial infarction which are categorized as modifiable (smoking, hypertension, high blood cholesterol, obesity, physical inactivity and diabetes) and non-modifiable (age, sex and family history of heart disease). Diabetes is a worldwide problem and is on the rise with age, obesity and decreased physical activity. The adult diabetes is predicted to increase from 2.8% in 2000 to 4.4% in 2030.^13^

In our study, 71.08% patients were thrombolysed within 12 hours of onset of chest pain, while 28.92% received thrombolytic after 12 hours. Those who were thrombolysed within 12 hours (i.e.59 patients), 43(72.88%) had complete resolution of ST elevation on ECG, 9 (15.25%) had partial resolution and 7 (11.86%) failed to resolve. Those who received thrombolytic after 12 hours (i.e. 24 patients), none of them had complete resolution, 12 (50%)had partial resolution and 12 (50%)failed to resolve. By applying chi-square test it gives us value of 36.470, with p-value <0.001, which is highly significant. This is supported by studies done in Shaikh Zayed Postgraduate Medical Institute, Lahore, Pakistan that mortality of the patient decrease by giving thrombolytic within 12 hours.^14^

In our study 28 patients were diabetic and the rest of 55 were non-diabetic. Out of 28 diabetics, 6 (21.43%) completely resolved their post thrombolysis ECG, 9 (32.14%) partially and 13 (46.43%) failed to resolve. On the other hand, in non-diabetics out of 55, 37 (67.27%) completely resolved as per ECG, 12 (21.82%) partially and 6 (10.91%) failed to resolve. Several studies have reported angiographic or ECG success in both diabetic and non-diabetic subjects, while others have shown that the diabetics have less complete resolution of ST elevation than the non-diabetics.^15^ On the other hand, in non-diabetics out of 55, 37 (67.27%) completely resolved as per ECG, 12 (21.82%) partially resolved and 6 (10.91%) failed to resolve. Thus in our study, complete resolution of ST elevation was observed more in non-diabetics as compared to diabetic patients (p value <0.001) This significant difference in ST-resolution between non-diabetics and diabetics was similar with the study done by Zairis et al. who showed significant difference between diabetic and non-diabetic patients in relation to complete (34.1% vs. 68.2%; p<0.001) and incomplete (65.9% vs. 31.8%;p<0.001) resolution.^16^ This can be explained by the fact that diabetics have increased levels of plasminogen activator inhibitor-1 and a procoagulent milieu that reflects poorer response of diabetics to thrombolysis.^17^

Out of those who were hypertensive (61), 26 (42.62%) had complete resolution and those who were non-hypertensive (22), 17 (77.27%)had complete resolution on ECG with a p-value of 0.019 that is also significant, thus indicating that hypertensive too have responded poorly to thrombolysis as compared to non-hypertensive in our study. However data on the impact of hypertension on ST resolution in patients +with ST elevation myocardial infarction (STEMI) are inconsistent and limited.

Dyslipidemia and site of infarction do not have a statistically significant effect on resolution of ST elevation post thrombolysis in STEMI patients according to the findings of our study.

## CONCLUSION

The results of our study suggest that patients with diabetes, hypertension and patients who receive thrombolysis after 12 hours of onset of chest pain respond poorly to thrombolytic therapy based on ECG findings whereas hyperlipidemias and site of infarction don’t affect the response of STEMI patients to thrombolysis.
